# Liquid Biomarkers for Improved Diagnosis and Classification of CNS Tumors

**DOI:** 10.3390/ijms22094548

**Published:** 2021-04-27

**Authors:** Severa Bunda, Jeffrey A. Zuccato, Mathew R. Voisin, Justin Z. Wang, Farshad Nassiri, Vikas Patil, Sheila Mansouri, Gelareh Zadeh

**Affiliations:** 1 MacFeeters-Hamilton Center for Neuro-Oncology Research, 4-305 Princess Margaret Cancer Research Tower, 101 College Street, Toronto, ON M5G 1L7, Canada; severa.bunda@uhnresearch.ca (S.B.); jeff.zuccato@mail.utoronto.ca (J.A.Z.); mvoisin@qmed.ca (M.R.V.); Justin.Wang@one-mail.on.ca (J.Z.W.); farshad.nassiri@mail.utoronto.ca (F.N.); Vikas.patil@uhnresearch.ca (V.P.); sheila.mansouri@uhnresearch.ca (S.M.); 2 Division of Neurosurgery, Department of Surgery, University of Toronto, Toronto, ON M5T 2S8, Canada

**Keywords:** liquid biopsy, ctDNA, proteomics, CSF, plasma, exosome, EV, CTC, microRNA

## Abstract

Liquid biopsy, as a non-invasive technique for cancer diagnosis, has emerged as a major step forward in conquering tumors. Current practice in diagnosis of central nervous system (CNS) tumors involves invasive acquisition of tumor biopsy upon detection of tumor on neuroimaging. Liquid biopsy enables non-invasive, rapid, precise and, in particular, real-time cancer detection, prognosis and treatment monitoring, especially for CNS tumors. This approach can also uncover the heterogeneity of these tumors and will likely replace tissue biopsy in the future. Key components of liquid biopsy mainly include circulating tumor cells (CTC), circulating tumor nucleic acids (ctDNA, miRNA) and exosomes and samples can be obtained from the cerebrospinal fluid, plasma and serum of patients with CNS malignancies. This review covers current progress in application of liquid biopsies for diagnosis and monitoring of CNS malignancies.

## 1. Background

The first description of fragmented circulating cell-free DNA (cfDNA) in human blood was published in 1948 [1] and the concept that individuals in various disease states have unique cfDNA profiles has been well studied since then [2]. It was recently determined that cancer patients have many analytes circulating in various biological fluids (biofluid), including blood, cerebrospinal fluid (CSF), urine, saliva and semen [3]. Specific analytes that have shown promise include circulating tumor cells (CTCs), circulating tumor DNA (ctDNA), exosomes, RNA, proteins and other metabolites that arise from their tumor [4,5,6,7,8]. Research on liquid biopsies, the non-invasive diagnosis and monitoring of cancer using biomarkers present in patient biofluids, has increased exponentially over the past decade. This has occurred, in part, due to the advent of highly sensitive methods for detecting and quantifying these analytes in biofluids [9]. While non-invasive biofluid collection for solid cancer diagnosis and subtyping is advantageous for all cancer patients, it arguably benefits most patients with central nervous system (CNS) tumors, due to differences in how diagnoses are currently being made for these patients.

Upon identifying a new CNS tumor on neuroimaging, the current practice is aimed at obtaining a tissue diagnosis with or without maximal safe tumor resection for cytoreduction [10]. These procedures have risks that can lead to neurological deficits in patients, significantly impacting their quality of life. Notably, a number of patients have tumors in eloquent areas of the brain, such as speech and motor regions, where the risk for neurological deficits can be greater [11,12,13]. These invasive biopsies are required since many different CNS tumor entities have similar appearances on neuroimaging and so cannot be diagnosed or subtyped without neuropathological assessment of tumor tissue. Furthermore, tumors located in the CNS originating from non-CNS sources, including CNS lymphoma and metastatic disease, are often indistinguishable from each other or from other primary CNS lesions [14]. Since neuroimaging cannot reliably discriminate metastatic lesions or more aggressive from more benign CNS tumors in many cases, management decisions regarding potential radiotherapy and systemic therapy use depend on the diagnosis made after surgery [14]. Specifically, more benign tumors may be managed with observation and serial neuroimaging after surgical biopsy. Therefore, a liquid biopsy could allow for a non-invasive diagnosis in these low-risk patients that avoids biopsy-related risks and informs subsequent management decisions without the need for upfront surgery.

**Figure 1:**
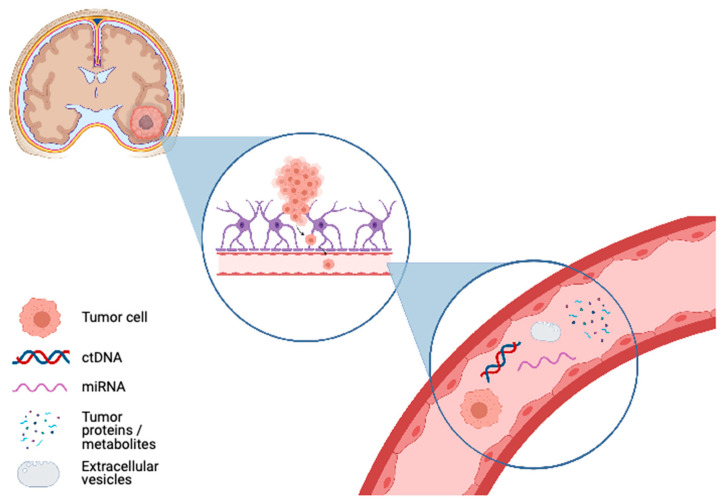
Liquid biopsy approaches utilized in central nervous sytem tumor diagnosis or monitoring. **Upper left panel**: CNS tumor of unknown diagnosis with local brain inflammation. **Middle panel**: Tumor analyte enters biofluid, with circulating tumor cell entering bloodstream and a representative example process is shown. **Lower right panel***:* analytes of interest depicted in the bloodstream as representative biofluid, including circulating tumor cells, circulating tumor DNA (ctDNA), microRNAs (miRNA), proteins, metabolites and exosomes/extracellular vesicles (EVs) (created with BioRender.com, accessed on 14 February 2021).

While the potential benefits of liquid biopsies for CNS tumors are well recognized, research in liquid biopsy approaches was first described in other major non-CNS tumor types that are not protected from systemic circulation by the blood-brain barrier (BBB). This microvasculature system of the CNS isolates the CNS from circulation, thereby leading to significantly lower amounts of circulating biomarkers [15,16]. Accordingly, approaches to CNS liquid biopsies are unique in that highly sensitive methodologies are required for their accurate detection and use in diagnostic or monitoring strategies. CSF is another source of circulating biomarkers related to CNS tumors that may enter directly through tumor invasion or through BBB dysfunction [17,18,19]. Figure 1 provides an overview of currently studied analytes for liquid biopsy approaches in CNS tumors. This review explores the current progress in CNS liquid biopsy research for each of the main biomarker types in blood, blood-related products, as well as CSF, and highlights the next major steps needed in order to refine and expand these techniques prior to implementation in future clinical practice.

## 2. Circulating Tumor Cells

Circulating tumor cells (CTCs) are a rare subset of cancer cells that depart from the solid tumor and enter a biofluid [20,21]. Although the presence of CTC may lead to disease progression, their detection can provide significant biological information regarding tumor burden and can be useful in clinical diagnostic and monitoring approaches, such as in non-invasive liquid biopsies for personalized cancer care [20]. CTCs were first described in common carcinomas including breast, prostate and colorectal carcinoma [22,23,24] and most recently in CNS malignancies [25]. Detection of CTCs via “liquid biopsies” is particularly important in malignancies of the CNS because of the potential to follow disease progression with a blood test, without repeat neurosurgical procedures that risk patient morbidity [25]. While major technological challenges exist in isolating viable CTCs due to their low biofluid abundance, many techniques have been developed and are under continuous improvement to enhance efficacy of CTC isolation [26,27].

Extracranial metastases from primary CNS tumors are rare events and this is thought to be due to the combined impact of the benign nature of many lesions, the aggressive nature and short survival of patients of high-grade brain tumors and the protective blood–brain barrier (BBB) [28,29]. Recent studies have shown the presence of CTCs in the CSF in greater than 10 adult glioma patients as well as in their blood, which suggests that tumor cells are capable of crossing the BBB and entering the circulation [30,31]. Many approaches have been utilized to detect glioblastoma (GBM) CTCs in the bloodstream, as a representative clinical entity which is the most common malignant primary brain tumor type. Notable approaches to their detection in GBMs include a telomerase promoter-based assay by Macarthur et al. in 11 glioma patients [32], antibodies directed against glial fibrillary acidic protein (GFAP), together with the detection of EGFR gene amplifications in 141 GBM patients by Muller et al. [33] and a microfluidic device by Sullivan and colleagues in 33 GBM patients [34]. Gao et al. identified CTCs in the blood from 24 of 31 patients (77%) within all subtypes of gliomas, including GBM, using an integrated cellular and molecular approach of SE-iFISH [35]. Bang-Christensen et al. also demonstrated that a recombinant malaria VAR2CSA protein (rVAR2) can be used for the efficient capture and detection of glioma cell lines, as well as glioma patient CTCs that are spiked into blood through binding to a cancer-specific oncofetal chondroitin sulfate [30]. The identification of CTCs in patients with various gliomas types suggested that the process of forming CTCs in the bloodstream across the BBB is not only limited to aggressive gliomas, but also occurs in more benign primary CNS tumors. Interestingly, using a commercially available CTC detection method, the Parsortix microfluidic technology, customized to detect both individual and multicellular clustered CTCs in an antigen-independent manner, Krol et al. demonstrated for the first-time the presence of CTCs in 13 GBM patients [29]. The presence of CTCs in gliomas has also been suggested to correlate with the recurrence rate of GBM and potentially to progression of lower grade gliomas, because of the capacity of CTCs to return to the tumor bed via a reseeding mechanism and repopulate the brain [36].

CTCs can also be isolated from the blood and CSF of pediatric brain tumor patients [37] and it has been shown that pediatric astrocytoma, ependymoma and medulloblastoma detection of CTCs [38] in the CSF is more efficient than in peripheral blood due to the disruption of the BBB [39]. Using a novel technique for the identification of CTCs utilizing an antibody specific to the cytoplasmic protein GFAP, bound to highly sensitive immunomagnetic liposome beads, Zhao et al. showed that CTCs can be successfully isolated from both the CSF and blood samples collected from 32 children with various brain tumors and that they are significantly more enriched in the CSF [40]. Although obtaining CSF necessitates a lumbar puncture that is more invasive than peripheral blood collection, these studies suggest that isolation of CTCs from CSF is more reliable than from patient blood using current methods and both may play a future role in the clinical diagnosis and treatment planning for brain tumors.

Brain metastasis occurs following the seeding of tumor cells from primary tumor arising outside of the CNS to the brain [41]. The process of brain metastasis is poorly understood and remains a significant cause of morbidity or mortality for patients with metastatic cancer. Dissemination of tumor cells from the primary tumor may result in their spread to the brain through the blood via CTCs. As such, CTC-based technologies can also be used to predict the pathway of metastasis and provide early diagnosis of metastatic disease to the brain from extracranial cancers [42]. To demonstrate tumor-initiating properties of CTCs, CTC-derived xenografts (CDX) were generated using CTCs isolated from patient blood of different cancer types, including breast, small cell lung cancer (SCLC) and non-SCLC (NSCLC) [43,44,45]. To identify a molecular signature of brain metastasis, Zhang et al. isolated CTCs from 38 breast cancer patients and showed that brain metastasis CTC signature comprising markers of HER2^+^/EGFR^+^/HPSE (human heparanase) ^+^/Notch1^+^ may be used to target brain metastasis-initiating CTCs [46]. They further characterized CTCs in metastatic breast cancer using gene expression profiling and showed that CTCs associated with brain metastasis have increased activity of the Notch signaling pathways, along with an increase of pro-inflammatory chemokines (TNF, IL1β and NF-κB), immunomodulatory networks (CXCL8, CXCR4, CD86) and mitogenic growth factors (PDGF-BB) [47]. Klotz et al. molecularly profiled CTC lines established from breast cancer patients and showed that copy-number gain of a mediator of blood–brain barrier transmigration, SEMA4D and overexpression of MYC may be novel markers for brain metastasis [42]. Ruan et al. analyzed the transcriptomes using single-cell RNA sequencing technology on CTCs isolated from CSF of five lung adenocarcinoma leptomeningeal metastases and identified metastatic-CTC signature genes enriched for metabolic pathway and cell adhesion molecule categories [48]. A comprehensive review describes recent research advancement of CTCs in brain metastasis [42] and this work has the potential to aid in preventative strategies in systemic cancer patients to reduce the risk of brain metastasis development.

The key advantage of assessing CTC levels and profiles in plasma and CSF of patients with CNS-related malignancies include its non-invasive nature and high sensitivity. However, the current limitation in utilizing CTCs in CNS malignancies are the technical limitations associated with isolation and enrichment of CTCs which are in low abundance in biofluids. Many techniques have been developed and are under continuous improvement to enhance the efficacy of CTC isolation and enumeration including approaches to limit cell loss and enhance cell selection and enrichment [27]. Furthermore, there are limited data sets available to demonstrate the clinical validity and utility of this technique. This approach is also relatively time-consuming, and the technical procedure is highly complicated. Additionally, the more recently developed microfluidic platform requires further validation that CTCs are indeed derived from GBM patients and it is overall an expensive technique.

Overall, it has been established that CTCs associated with CNS cancers can be detected in both blood and CSF and work is ongoing to potentially implement this work into clinical practice. The identification of CNS tumor molecular alterations within CTCs via liquid biopsies has the potential for significant future clinical utility enabling tumor diagnosis, the identification of susceptibility to available targeted treatments, and monitoring disease progression [25].

## 3. Circulating Tumor DNA

Circulating tumor DNA is a subset of all cell-free DNA (cfDNA) and the product of CTCs undergoing apoptosis as well as CNS tumor nucleic acids entering biofluids by crossing the BBB to enter the bloodstream [49]. ctDNA is observed as 150–200 nucleotide size fragments [50]. Due to low ctDNA concentration in biofluids, methods of its detection are often highly specific but lack sensitivity, leading to low false positive rates and high false-negative rates. This is a particular problem in plasma samples where it is estimated that ctDNA constitutes <1% of all cfDNA, particularly early in cancer development when disease burden is typically lower [50]. However, as ctDNA detection methods continue to evolve and improve, the sensitivity is increasing [51]. Current main approaches to diagnose, prognosticate, identify therapeutic targets within and monitor treatment response/recurrence of CNS tumors using ctDNA include the identification of genomic alterations and epigenetic signatures.

Many next-generation sequencing (NGS) and PCR-based methodologies have been utilized in CNS tumors to identify genetic markers, in particular, those of gliomas [52]. Droplet digital PCR (ddPCR) is a notable PCR-based technique for these applications as it is able to detect and quantify targets with low copies at high sensitivity. This technique uses water-emulsion droplet technology to fractionate a DNA sample into approximately 20,000 droplets, which then individually and independently undergo PCR amplification for precise target quantification [53]. Within tumor samples, ddPCR assays are capable of detecting mutations down to 1/100,000 copies or 0.001% [54]. This technique becomes invaluable when working with liquid biopsy cfDNA samples containing low quantities of ctDNA, especially in plasma samples with less than 1% ctDNA and 99% non-tumor DNA.

ddPCR has been shown to accurately detect alterations of intertest in non-CNS tumor blood/plasma samples including BRAF V600E alterations in melanomas and EGFR mutations in non-small cell lung cancer, both have targeted treatments available [55,56]. ddPCR is also highly sensitive and specific compared to Sanger sequencing as the gold standard for IDH mutation identification in glioma tumor tissue samples, with the ability to detect these IDH mutations with 100% sensitivity and 96% specificity to guide patient diagnosis [53]. A study of 127 plasma samples from 41 pediatric diffuse midline glioma patients by Izquierdo et al. obtained 5.3 ng of cfDNA per mL of plasma used and utilized a mean of 1.8 ng of cfDNA for ddPCR. None of the plasma samples were positive for known alterations but the cfDNA concentration was higher in radiated patients, potentially due to BBB disruption and ctDNA release (NOA vdab013). Although the yield of cfDNA is only marginally higher in non-CNS tumor plasma (6.0 vs. 5.3 ng of cfDNA per mL of plasma), ~99% of cfDNA is non-tumor DNA and so large differences in the abundance of the remaining 1% of cfDNA in CNS vs. non-CNS tumors are not apparent when comparing total cfDNA values [57].

Because of the ability to quantify the the absolute number of mutation copies present, ddPCR has also recently been used to monitor disease progression and predict response to treatment in blood samples of non-CNS cancers. A recent study from Forthun et al. in metastatic malignant melanomas used ddPCR mutation assays to quantify BRAF and NRAS ctDNA alterations present in order to prognosticate 50 patients and predict response to treatment [58]. Unfortunately, there is a limited presence of cfDNA in the plasma of CNS tumor patients due, in part, to the BBB, so results from ddPCR in CNS tumor plasma is less robust. In García-Romero et al.’s cohort of 29 pediatric patients with medulloblastomas, ependymomas, or gliomas, the accuracy of BRAF V600E mutation detection using ddPCR on cfDNA extracted from 500 uL of plasma was low (sensitivity 25%, specificity of 78%) compared to tumor mutation testing [59]. However, a recent study by Muralidharan et al. examining TERT promoter mutations in plasma samples from 157 adult glioma patients showed a much higher utility of ddPCR to detect these alterations, with a sensitivity of 62.5% and a specificity of 90%. Notably, during longitudinal monitoring of 5 of these patients, those with tumor recurrence had increased mutant allele frequencies compared to those without recurrence suggesting that ddPCR may have a future role for detecting disease progression [60].

Approaches to improve the detection capabilities of ddPCR are being assessed including pre-amplifying cfDNA prior to ddPCR [61] and utilizing high affinity locked nucleic acid-enhanced probes to reduce the formation of secondary structures and stabilize amplification [60]. Sampling ctDNA through cerebrospinal fluid instead of plasma is an emerging approach to avoid the non-tumor contributors to plasma cfDNA while also accessing a biofluid with a higher ctDNA concentration [52]. Accessing ctDNA in CSF samples of brain tumor patients have shown significant promise in many CNS cancer types [62,63,64]. Overall, it should be noted that while ddPCR is a low-cost, simple, and highly specific method, it offers relatively low sensitivity for detection of alterations in liquid biopsy samples and is limited to the assessment of only specific alterations.

The assessment of epigenomic signatures in the form of DNA methylation alterations across the genome is not limited to the assessment of specifically known alterations and this is a major advantage when profiling low amounts of ctDNA. The main approaches to evaluate DNA methylation patterns in tumor tissue samples directly include whole-genome bisulfite sequencing (WGBS)), as well as reduced representation bisulfite sequencing (RRBS). Methylation signatures identified in tumor samples are highly tissue-specific and useful for CNS tumor diagnosis [65,66]. The main methodology used to evaluate ctDNA methylomes in CNS tumor patients is termed the cell-free methylated DNA immunoprecipitation combined with deep sequencing approach (cfMeDIP-seq). This technique utilizes small quantities of input cfDNA (1–10 ng) and recapitulates the same methylation patterns to those obtained by WGBS and RRBS when using 2000 ng and 1000 ng, respectively. Additionally, cfMeDIP-seq shows enrichment for regions of CpG islands which are highly relevant for distinguishing cancer types [67].

The cfMeDIP-seq protocol involves an immunoprecipitation and subsequent sequencing of methylated cfDNA obtained from biofluids. Unique features of this protocol that address the issue of low input amounts are the utilization of filler DNA that acts as a carrier for immunoprecipitation of small cfDNA volumes, along with the amplification and size selection of final libraries to limit sequencing to those ctDNA fragment sizes of interest in the 150–200 bp range. The cfMeDIP-seq approach has been used by Nassiri et al. to detect CNS tumor plasma methylomes that allow for many brain tumor types to be distinguished with high accuracy including IDH mutant gliomas, IDH wildtype gliomas, meningiomas, hemangiopericytomas, and low grade glioneuronal tumors [68]. The bioinformatics approach to distinguish these tumors involves the development of ensembles of classification models comparing one tumor type to all others using differentially methylated regions of the plasma methylome. The performance of these models in the testing set samples not used to develop the models are assessed with mean areas under receiver operating characteristic curves (AUROC) representing model accuracy between 0.7 and 1.0 for models discriminating these CNS tumor types in this study. Specific mean AUROCs per tumor type were 0.82 (95% CI = 0.66–0.98) for IDH mutant gliomas, 0.71 (95% CI = 0.53–0.90) for IDH wildtype gliomas, 0.89 (95% CI = 0.8–0.97) for meningiomas, 0.95 (95% CI = 0.73–1.00) for hemangiopericytomas and 0.93 (95% CI = 0.8–1.0) for low grade glioneuronal tumors. While this study demonstrated the ability to differentiate IDH mutant from IDH wildtype gliomas, unfortunately further molecular subtyping of diffuse gliomas, including 1p19q codeletion status was not included as independent groups. The results of cfMeDIP-seq use in CNS tumors are of similar efficacy to those seen in using plasma methylomes from cfMeDIP-seq to discriminate non-CNS cancer types [69,70] and does not appear to be significantly affected by the lower plasma ctDNA content in CNS tumor patients due to the BBB.

Although the low ctDNA content limits the utility of WGBS or RBBS for non-invasive CNS tumor diagnosis, one recent study by Sabedot et al. used 2–100 ng of serum ctDNA for WGBS and showed efficacy in discriminating and monitoring gliomas [71]. Using 38 glioma samples and 42 non-glioma control samples the authors developed a glioma epigenetic liquid biopsy score called GeLB that was highly accurate is detecting gliomas versus non-glioma patient with a sensitivity of 100% and specificity of 97.8%. This score also showed utility in monitoring disease progression and response to therapy. Additionally, WGBS of ≤10 ng of ctDNA obtained from 3 patient CSF samples have been used by Li and colleagues for the non-invasive detection of medulloblastomas versus normal controls but in 1 (of 4) patient samples had low data quality led to sample exclusion from the analysis [72].

Multiple approaches have been utilized to assess CNS tumor genomic alterations and DNA methylation patterns using ctDNA from plasma for the purposes of tumor diagnosis and monitoring. ddPCR has shown high specificity for genomic alterations in CNS tumors and approaches to improve its sensitivity are currently being assessed including approaches to optimize results in plasma samples as well as the utilization of CSF as the ctDNA source. Plasma cfMeDIP-seq has proven to be both a highly sensitive and specific methodology for the discrimination of major CNS tumor types. Further work to detect other clinically meaningful subtypes including 1p19q co-deleted gliomas and aggressive meningiomas and further refinement of this technology for clinical practice is needed. Altogether, assessment of whole-genome DNA methylation using cfMeDIP-seq offers high sensitivity, requires low input DNA, and genome-wide assessment of DNA methylation can be performed in a single test. However, this approach is more costly than ddPCR which examines specific alterations of interest.

## 4. Circulating Tumor MicoRNA

microRNAs (miRNAs) are endogenous, non-coding RNAs that are 18–22 nucleotides long and primarily modulate gene expression at the post-transcriptional level through a combination of translational repression and mRNA destabilization [73]. As a result, miRNAs regulate the majority of known biological processes including cell proliferation, differentiation, metabolism and development [73]. Thus, deregulation of miRNA expression plays a role in cancer initiation and progression [74]. Furthermore, miRNAs can function either as a tumor suppressor or oncogenic. Therefore, changes in a single or small subset of miRNAs can modulate several cellular processes and lead to tumorigenesis [74]. There are two main types of circulating miRNAs: vesicle-packaged miRNAs and miRNAs complexed with the Argonaute-2 proteins, both of which are cell by-products that accumulate in extracellular fluids [75]. The majority of existing studies on circulating miRNAs in the blood as non-invasive biomarkers for tumors have focused on the entire circulating pool of miRNAs instead of vesicle-packaged miRNAs. One of the advantages of utilizing circulating miRNAs as biomarkers is their greater stability relative to mRNAs and therefore prolonged storage at room temperature along with freezing or thawing have minimal effects on miRNA expression levels [76].

Widespread technologies used to measure miRNA expression in biological samples include microarray, next-generation sequencing (NGS) and quantitative real-time PCR (qPCR). Microarray-based miRNA measurement platforms provide overall miRNA expression profiles with reasonable cost and throughput. Nevertheless, although qPCR is more expensive per sample and has a lower throughput, the amplification technology offers much higher sensitivity than microarrays [77]. NGS can identify new miRNAs, is cheaper than microarray or qPCR, and requires small amounts of samples even though it is tedious and has a very long handling time. Therefore, qPCR remains the top choice for validation and clinical analysis of large numbers of samples.

Using these high-throughput techniques, several miRNAs have been identified and reported as potential circulating biomarkers for tumors in blood and blood product derivatives. Circulating miRNAs identified with altered patterns of expression may aid with tumor diagnosis and patient prognostication in gliomas as well as other CNS tumors, but currently, the majority of these findings have not been validated in additional cohorts [78]. Firstly, Wang et al. showed that levels of miR-21 are significantly upregulated, while miR-128 and miR-342-3p are down-regulated, in the serum of patients with GBM patients compared to healthy controls [79]. While downregulation of miR-342-3p was confirmed in a separate study, miR-128 was found to be up-regulated in the plasma of GBM patients and so the result for this potential biomarker were not consistent [80]. In a separate study, combined analyses of miR-21 and miR-15b expression allowed for discrimination of glioma patients from healthy individuals [81]. In a case-control diagnostic study, Ohno comprehensively assessed 580 serum samples and 2565 miRNAs, including samples from 157 patients with diffuse glioma and developed a glioma index that discriminated diffuse gliomas from noncancer controls and the 3-Tumor Index, which discriminated GBMs from primary nervous system lymphomas (PCNSL) and metastatic brain tumors [82].

Additional studies have identified prognostic miRNA signatures. Wang et al. also observed that the expression of miR-128 and miR-342-3p positively correlated with glioma grade [79]. In addition to gliomas, Zhi and colleagues reported that upregulated serum levels of miR-20a, miR-106a, and miR-181b are associated with advanced clinical stage of astrocytoma [83]. Another study found that lower serum levels of miR-497 and miR-125b [84] and miR-29 [85], can be used to distinguish high grade from low-grade gliomas [84]. Zhang and colleagues [86] demonstrated a significant upregulation of miR-221/222 family miRNAs in the plasma of glioma patients and a higher expression of these miRNAs correlated with poorer overall survival in this series. Circulating miRNAs may also be used as biomarkers of recurrence as demonstrated by decreased levels of serum miR-21 after tumor resection [85,87] and by its increased expression upon tumor progression [85].

These examples clearly show that discovering a sensitive and specific miRNA signature for CNS malignancies is promising but work is ongoing and there are challenges to overcome. The lack of clinical miRNA biomarkers compared to the number identified in the research is likely due to limitations in standardizing preanalytical features such as inconsistency in study design and tumor types studied sample type (serum vs plasma, whole blood vs exosomes), miRNA coverage, and analytical techniques. Furthermore, extensive heterogeneity and a variety of subtypes of gliomas, may account for variability across different studies. Nevertheless, a meta-analysis performed by Qu and colleagues to assess the diagnostic value of miRNAs revealed miR-21 as the most powerful and reproducible clinical biomarker in diagnosis of GBM [88]. In addition, Zhou et al. reviewed 28 articles investigating miRNA-based glioma diagnosis and reported overall sensitivity of 85%, specificity of 90%, and AUC of 93% for these models [89].

Little is known about circulating miRNAs and their clinical utility in other CNS tumors. Zhi et al. performed a TLDA assay to assess the serum miRNA expression profile in 20 meningiomas and 20 healthy controls [90]. Candidate miRNAs were subsequently validated in 210 patients and 210 healthy controls from two independent cohorts by qRT-PCR. The authors found that the serum levels of miR-106a-5p, miR-219-5p, miR-375 and miR-409-3p were significantly higher, while the levels of miR-197 and miR-224 were markedly lower in meningioma patients compared to healthy controls. Notably, they also demonstrated that the level of a subset of these miRNAs correlated with tumor load, patient sex, the clinical stages of meningioma and tumor recurrence rates. They finally proposed a panel of 6 serum miRNA that may be utilized as an auxiliary clinical tool. Another study assessed serum microRNAs as a possible biomarker of brain metastasis (BM) in patients with breast cancer and discovered that serum miR-4480 and PgR negative are useful biomarkers for predicting BM in patients with breast cancer [91]. Therefore, serum miR-4428 could be a biomarker and further investigation might be warranted.

While the majority of studies have assessed miRNA expression in serum, a less studied source of miRNA biomarkers is plasma. Several reports have raised the prospect that plasma and serum might exhibit differences in their miRNA content, suggesting that different blood sample preparation methods might affect the concentration of individual circulating miRNAs [92]. It is well documented that the coagulation process increases sample-to-sample variations which complicates data analysis, suggesting that plasma may be a more appropriate source of circulating miRNA, since RNA released during the coagulation process may change the true repertoire of circulating miRNA. Specifically, when assessing archived samples, special attention must be given to this difference as the majority of the archived samples are stored as serum.

CSF has shown early promise as a biofluid for the detection of miRNAs and its isolation from general circulation suggests that it may be a more specific and accurate source of circulating miRNAs in comparison to blood products. Baraniskin et al. identified miR-21 and 15b in the CSF of patients with malignant glioma [93]. Another pilot study [94] identified a miRNA signature that could potentially be used to diagnose or discriminate GBM from brain metastases. Although not as well studied as miRNAs in blood, CSF is a promising source of miRNAs for the diagnosis of gliomas and potentially to differentiate glioma subtypes [95].

While the discovery of miRNAs in biofluids offers a new perspective on their utility as disease biomarkers, circulating miRNAs have failed to enter clinical practice due to inconsistent and irreproducible findings. Therefore, before circulating, miRNAs can reliably be used as biomarkers of disease, analytical factors that may affect their measurement, including sample type, measurement platform, or normalization strategy need to be addressed. Major pre-analytical variables affecting circulating miRNAs have been characterized [96]. Mechanical hemolysis, caused by improper blood collection or preparation, could alter the level of circulating miRNAs through contamination by intracellular miRNAs. Regardless of the platform used for miRNA measurement, data normalization to correct for variability during sample preparation, and to quantify miRNA expression is another major issue. The addition of spike-in standards prior to cDNA synthesis and PCR can estimate efficiency and normalize the results for comparison [77]. Application of digital PCR for measurement of miRNAs could circumvent normalization issues since it is an absolute method of nucleic-acid quantification. As such, droplet digital PCR (ddPCR) is a highly precise method and does not rely on reference standard curves and has recently been shown to reduce analytic variability and to improve day-to-day reproducibility compared to RT-qPCR [77].

## 5. Circulating Proteome

Proteomic profiling has recently become an active area of research for biomarker discovery and the identification of new therapeutic targets. Recent studies have shown that specific proteomic patterns can differentiate subtypes or grades of CNS tumors [97,98,99]. Modern technological advancements, which allow rapid screening, low input sample, and accurate protein identification, have enhanced the accuracy of proteomic analyses and will likely accelerate CNS tumor biomarker discovery [100].

One promising source for protein biomarker discovery is the CSF, where protein presence may result from either secretion/leaking by tumor tissues or the disruption of the BBB [19]. A number of reports have described the analysis of the CSF proteome in different CNS malignancies. For example, the CSF level of carcinoembryonic antigen (CEA) was found informative in the differential diagnosis of primary and metastatic brain tumors [101,102] and applicable auxiliary marker for the diagnosis of meningeal carcinomas [103,104]. In a study by Khwaja et al., the authors reported that proteomic analysis of CSF can discriminate malignant and non-malignant CNS diseases and identified carbonic anhydrase protein as a prognostic marker of brain cancer [105].

A large body of work on CSF proteins that have impacted the clinical management of CNS cancer has been performed on intracranial malignant germ cell tumors [106]. Germ cell tumors maintain the molecular profile of their primordial lineage as they retain the expression of embryonic proteins, such as beta-human chorionic gonadotropin (bHCG) and alpha-fetoprotein (AFP) [107]. These proteins are prominently elevated in the CSF of intracranial malignant germ cell tumor patients [108] and are currently utilized clinically as diagnostic and precise indicators of response to therapy. In fact, analysis of both markers in the serum or CSF is required in order to distinguish between germinoma and NGGCT non-germinoma germ cell tumors and it is used to stratify patients into high risk for more intensive treatment and to assess the risk of tumor recurrence. Other, less specific markers, such as placental alkaline phosphatase (PLAP) and lactate dehydrogenase isoenzymes, were found to be clinically useful in the diagnosis and monitoring response to therapy of pediatric intracranial germinomas [109]. Elevated levels of s-kit, the soluble form of the c-kit receptor, were found to be reliable for germ cell tumor diagnosis and to differentiate germ cell tumors from other CNS malignancies. Miyanohara et al. also reported that s-kit expression is able to detect the recurrence of germ cell tumors and their subarachnoid dissemination [110].

The majority of studies to date have focused on the identification of circulating proteomic biomarkers in the CSF of GBM patients. Shen et al. conducted a review of the literature and determined an increased level of 19 proteins, while one protein (GSN) was downregulated in the CSF of glioma patients and might be involved in glioma pathogenesis [19]. On the same theme, Khwaja et al. used two-dimensional gel electrophoresis and cleavable Isotope-Coded Affinity Tag to identify tumor- and grade-specific proteomic biomarkers in the CSF [18]. Their retrospective analyses on 60 samples (World Health Organization [JG1] grades II, III and IV astrocytomas, schwannomas, metastatic brain tumors, inflammatory samples, and non-neoplastic controls) the authors identified 103 tumor-specific markers, 20 of which were specific to high-grade astrocytomas. Sampath et al. investigated CSF samples from 27 patients with high-grade astrocytomas, 39 patients with non-astrocytic CNS neoplasms and 14 patients with no known CNS neoplasm and found that VEGF was detectable in the CSF of 89% of patients with malignant astrocytoma and was absent in normal CSF samples [18]. The levels of VEGF were significantly higher in high-grade astrocytomas than in non-astrocytic tumors indicating that detection of VEGF in CSF could be a potential marker for differentiating astrocytic tumors. Using mass spectrometry Schuhmann et al. identified four CSF peptides which significantly distinguished GBM from controls [111].

Diffuse intrinsic pontine glioma (DIPG) is a pediatric CNS tumor that is not surgically resectable, resulting in a paucity of tissue available for molecular studies and, currently, there are no effective treatments. Saratsis et al. [112] used mass spectrometry to assess the proteome of 15 CSF specimens collected from patients with DIPG showing selective upregulation of Cyclophilin A (CypA) and dimethylarginase 1 (DDAH1) in DIPG compared with controls, suggesting that detection of these factors in CSF and serum has potential clinical application, with implications for assessing treatment response and detecting tumor recurrence in patients with DIPG.

Proteomic analysis of CSF has revealed various proteins that are differentially expressed in CNS lymphomas [113,114]. For example, antithrombin III (ATIII) is a serine protease inhibitor that is associated with neovascularization in CNS lymphoma and has been prospectively validated [115]. ATIII expression was reported by Roy et al. to be elevated in the CSF of patients with CNS lymphoma compared to healthy control [114]. Further, elevated ATIII levels significantly correlated with shorter survival rates and less responsive to chemotherapy. However, other studies claim that ATIII is not a suitable biomarker for the diagnosis of PCNSL and increased concentrations of ATIII in CSF might be due to leakage of the BBB [114,116]. CXCL13 protein that is known to mediate chemotaxis of CNS lymphoma cells was detected within biopsy specimens from PCNSL patients [117] raising the possibility that this chemokine may contribute to CNS tropism. Rubenstein et al. [118] investigated the concentration of CXCL13 in CSF of CNS lymphoma patients and control cohorts in a multicenter study involving 220 patients. Their result demonstrated that elevated concentration of the chemokine CXCL13 concentration in CSF is a highly specific marker for the detection of CNS lymphoma and can be helpful as an adjunctive diagnostic test and response to treatment assessment. In addition to CXCL13, Sasagawa et al. [119] reported that the level of IL-10 in CSF is a superior biomarker for initial screening of patients with CNS lymphoma compared to CXCL13.

Medulloblastoma (MB), the most common malignant brain tumor in children includes various subtypes with group 3 and 4 subtypes being clinically distinct with regard to metastasis and prognosis, which may also manifest in a difference in their proteomic spectra. Rajagopal et al. investigated the CSF proteome from 33 children with MB compared to 25 age-matched controls using 2D- gel electrophoresis and found that levels of prostaglandin D2 synthase (PGD2S) was six-fold lower in the CSF of tumor samples suggesting a host response to the presence of the tumor [120,121]. Biomarkers are often thought to be elevated in a disease state compared to normal levels, however, candidate negative diagnostic marker such as PGD2S could be useful for detecting MB as well as recurrence of the disease. On the other hand, while negative biomarkers are potentially useful, their relationship to tumor biology is less direct and more highly complex in comparison to proteins that are over-expressed in tumor-associated samples [106]. Desiderio et al. performed proteomic profiling of CSF from 14 children with posterior fossa tumors (6 Pilocytic astrocytoma, 5 Medulloblastoma, 3 Ependymoma and 5 nontumoral control) and demonstrated that the hemoglobin subunit beta fragments (peptides LVV- and VV-hemorphin-7) could serve as a biomarker in posterior cranial fossa pediatric brain tumors [122]. Both LVV- and VV-h7 were detectable in control CSF but absent in the patients’ CSF collected before surgery (i.e., in presence of tumor). Their data suggest that analysis in post-surgery CSF could be used to predict patient prognosis. Finally, levels of polysialic-neural cell adhesion molecule (PSANCAM), considered a marker of developing neuron were found to be significantly higher in CSF from MB patients that are refractory to treatment or those who relapsed than patients in remission [123].

Although CSF has been a well-studied biofluid for CNS tumor circulating proteins, blood product biomarkers have also been studied. Atypical teratoid/rhabdoid (AT/RT) tumor is a rare malignant CNS tumor commonly found in children less than 5 years of age. Osteopontin (OPN) a bone matrix glycoprotein levels were found to be significantly elevated in patients with AT/RT. Using enzyme-linked immunosorbent assay and immunohistochemical analysis, Kao et al. investigated plasma, CSF, and brain tissue specimens from 39 patients MB, 16; AT/RT, 8; epilepsy, 6; hydrocephalus, 9 and found that patients with AT/RT have higher plasma and CSF OPN levels in comparison to the rest of the cohort [124]. Interestingly, a significant correlation between OPN levels and the risk of tumor relapse was identified and OPN levels in the CSF were found to decrease with treatment.

Overall, research on traditional sampling sources for proteomic profiling including CSF and blood products [100,125] as well as tissue lysates [126] have yielded a substantial amount of information on potential brain cancer biomarkers. However, the value majority of these markers in a clinical setting remain unclear and there is a significant need for further work in studying proteomic biomarkers in the bloodstream as the they are less invasive to obtain and more translatable into clinical practice.

## 6. Extracellular Vesicle

Extracellular vesicles are membrane-bound vehicles secreted by cells that mediate intercell communication, membrane remodeling, recycling, and removal of cellular components [37,127,128]. A variety of molecules can be encapsulated within EVs from their parent cell including but not limited to nucleic acids (DNA, mRNA, miRNA, LncRNA, circRNA), proteins, and lipids. The study of EVs as liquid biopsy biomarkers has gained popularity recently with RNA, proteins, and microRNA (miRNA) being the most commonly studied macromolecules of interest [129]. The types of extracellular vesicles include exosomes (30–150 nm in size), microvesicles (50–2000 nm in size), retrovirus-like particles (90–100 nm), apoptotic bodies (50–4000 nm), and more. Although clear size cut-offs and unique surface markers that distinguish these extracellular vesicles (EV) populations from one another do not currently exist, their isolation and analyses may differ e.g., flow cytometry often cannot analyze extracellular vesicles smaller than 200 nm whereas nanoparticle tracking or electron microscopy can [130].

EVs hold several advantages over other liquid biopsy analytes. To start, EVs exist in nearly all bodily fluids including blood, plasma, cerebrospinal fluid (CSF), ascites, semen, saliva, and urine [129,130,131,132,133,134,135]. Additionally, their biological stability ensures they can be stored at a variety of temperatures without degradation of their contents [129]. Furthermore, because EV contents are derived from living parent cells, they may be more representative of true biological processes compared to circulating ctDNA which is often derived from apoptotic cells [136]. EVs can also express surface proteins specific to their parental cell of origin which can enable investigators to isolate organ- or tumor-specific exosomes and even predict organ-specific metastases [137]. Lastly, the sensitivity and specificity of exosomal DNA have been found to be greater than for ctDNA in terms of detecting mutational frequency and potentially also in prognosticating patients [138,139].

CNS tumors, due to their location behind at least a partially intact BBB, do not often release the same quantity of CTCs and soluble proteins as their non-CNS counterparts as described above [140]. They do, however, release cellular components within EVs that have been exploited for liquid biopsies. To date, the majority of studies on EVs as a liquid biopsy biomarker have been on glioblastomas. Chen et al. analyzed EV mRNA isolated from the CSF of glioma patients using a form of digital PCR termed BEAMing (Beads, Emulsions, Amplification, Magnetics) and ddPCR. They were able to identify the prognostically important mutant IDH1 mRNA in CSF-derived EVs from five of eight patients with IDH1-mutant tumors, but not in their matched serum-derived EVs. Additionally, they were able to quantify the number of mutant IDH1 transcripts, which directly correlated with tumor burden [141]. Up to one-third of all GBMs harbor the epidermal growth factor receptor *(EGFR) vIII* mutation resulting in a constitutively active EGFR that does not bind its ligand, but rather, promotes tumor proliferation through downstream events [142]. An early study by Skog et al. found the *EGFRvIII* status of the original tumor was recapitulated in the patients’ serum EVs when examined by qRT-PCR. However, this was only seen in five of 14 patients with biopsies positive for the *EGFRvIII* mutation, suggesting either suboptimal sensitivity of serum EVs and/or sampling error in the original tumor biopsies due to heterogeneity. Interestingly, on longitudinal serum analysis in those five patients with the *EGFRvIII* mutation seen in serum EVs, *EGFRvIII* was undetectable after tumor resection, again suggesting a role for serum EVs in monitoring treatment outcome and disease burden [143]. In a later study, Figueroa et al. was also able to detect the *EGFRvIII* mutation in the EV of 14 of 23 GBM patients, albeit in the CSF this time with 60% sensitivity and 98% specificity, compared to the gold standard qPCR detection in tumor tissue [143].

The microRNA miR-21 has been implicated in GBM pathogenesis through its effect on a variety of pathways including IGFBP3, RECK and TIMP3 that mediate a number of oncogenic functions such as suppression of apoptosis, growth proliferation, and DNA repair [144]. Akers et al. demonstrated that GBM cells actively secrete EVs with miR-21 and that CSF levels of miR-21 EVs were 1-fold higher in GBM patients than in non-oncologic controls. They were able to prospectively differentiate between an independent cohort of 29 GBM patients and non-oncologic patients prospectively, suggesting a role for this particular biomarker to be used for future screening and demonstrated a significant decrease in miR-21 levels following surgical resection of a patients’ GBM [127]. The diagnostic value of miR-21 is also discussed in the miRNA section of this article.

Shao et al. pioneered a micro-fluidic chip that enabled sensitive detection of microvesicle number and protein expression in blood samples from GBM patients by labeling microvesicles with target specific magnetic nanoparticles and imaging them using a miniaturized micronuclear magnetic resonance system. They demonstrated that the sensitivity of their technique exceeded other contemporary methods of protein detection including Western blotting and ELISA in measuring EGFR, EGFRvIII, podoplanin, IDH1 R132H molecules in EV isolated from the serum of 24 GBM patients and eight healthy controls. They also found that patients with a higher quantity of tumor-associated molecules (e.g., EGFR, EGFRvIII, podoplanin) in their EVs were more likely to fail standard of care therapy with chemoradiation (radiotherapy plus temozolomide) [130,140]. 

As with any liquid biopsy approach, true validation of this technique requires a well-characterized cohort of healthy patients without radiographic evidence of CNS disease that are age-matched to patients with brain tumors. It also remains unclear whether EVs in the CSF have greater diagnostic yield compared to EVs in other bodily fluids such as plasma or serum. Particularly for CSF, there are not yet guidelines for its storage and analysis for the purposes of extracting EVs, nor have various canonical EV biomarkers been characterized. Current analyses of EVs remain predominantly one-dimensional using dynamic light scattering instruments such as Nanosight, but more multi-parametric analysis tools with improved resolution and discriminative capabilities are being developed and increasingly used [145]. Analyzing the spatial distribution of a target molecule within or around an EV of interest is also currently limited as there are no well-established counterstain equivalents used in the immunofluorescence imaging of adherent cells as EVs generally lack a nucleus or nuclear content and even an actin cytoskeleton. The use of orthogonal techniques such as transmission electron microscopy or mass cytometry may help address or mitigate these limitations [146].

Given the clear clinical need for minimally invasive methods of diagnosis and tracking of treatment response in CNS tumors, EVs offer promise as a biomarker for CNS tumors and they offer the benefit of containing multiple encapsulated nucleic acids and proteins for analysis. However, further investigation in other CNS tumor types, as well as additional research using larger sample sets and prospective clinical trials, are needed before the routine clinical application can be considered. 

## 7. Future Directions

The molecular assessments of circulating tumor cells, nucleic acids, proteins and metabolites, and exosomes have each shown promise to contribute to non-invasive CNS tumor detection for the purposes of diagnosis and monitoring. Currently, these approaches have been studied individually for the most part and have not yet been implemented into standard of care neuro-oncology practice. The currently active next steps to optimize liquid biopsy approaches are focused on improving the utility of the methodologies used to detect each of these tumor analytes in blood and CSF, as well as expanding the number of molecular and pathological subtypes able to be detected that could aid future clinical decision-making. It will be important for these techniques to be validated in additional prospective cohorts to further establish their utility, with a focus on the sensitivity and specificity of each technique. Following the optimization of these methodologies, future work combining multiple analytes into comprehensive molecular tests may optimize the sensitivity and specificity of liquid biopsies for CNS tumor diagnosis and monitoring by combining data from multiple platforms. It is expected that non-invasive molecular profiling of CNS tumors maybe implemented into clinical practice in the future to transform patient care by allowing for non-invasive diagnosis of CNS tumors to avoid surgical risks and allow for early detection of tumor development and progression.

## Data Availability

Not applicable.
